# Pulmonary vein isolation using a novel balloon-in-basket pulsed field ablation system: The Lübeck how-to protocol

**DOI:** 10.1016/j.hroo.2026.02.022

**Published:** 2026-03-06

**Authors:** Roland Richard Tilz, Sascha Hatahet, Charlotte Eitel, Sorin Popescu, Behnam Subin, Karl-Heinz Kuck, Roman Mamaev, Jan-Per Wenzel

**Affiliations:** 1 Department of Rhythmology, University Heart Center Lübeck, University Hospital Schleswig-Holstein, Lübeck, Germany; 2 German Center for Cardiovascular Research (DZHK), Partner Site Hamburg/Kiel/Lübeck, Lübeck, Germany

**Keywords:** Pulmonary vein isolation, Atrial fibrillation, Pulsed field ablation, Balloon-in-basket, Workflow, Learning curve

## Abstract

**Background:**

Single-shot pulsed field ablation (PFA) is increasingly used for pulmonary vein isolation (PVI) in atrial fibrillation (AF). Although balloon-in-basket (BiB)-PFA system integrates ablation and mapping into a single catheter, standardized procedural workflows remain limited.

**Objective:**

This study aimed to describe a standardized, step-by-step workflow for AF ablation using the BiB-PFA system and evaluate procedural performance, acute safety, and the learning curve in clinical practice.

**Methods:**

This prospective, single-center study included 40 consecutive patients undergoing index PVI using the BiB-PFA system at a tertiary high-volume electrophysiology center. Procedures followed standardized protocol including balloon preparation, ultrasound-guided access, fluoroscopy-guided transseptal puncture, selective pulmonary vein (PV) angiography, and a structured 2-step ablation approach for each vein. Posterior wall ablation (PWA) was performed in selected patients. Procedural metrics and acute complications were analyzed descriptively.

**Results:**

Patients were 64.5 years old (56.5, 73.3); 41% were female, and 57.5% had nonparoxysmal AF. The median procedural duration was 41.5 minutes (35.0, 59.0), left atrial dwell time 27.5 minutes (25.0, 36.0), and BiB dwell time 23.0 minutes (20.0, 25.8). A median of 4 applications per PV was used. Reduced-energy delivery was required in 30% of right inferior and 25% of right superior PV. PWA was performed in 35% of patients using a median of 5 applications. No major intraprocedural complication occurred. The mean procedural duration decreased from 50.0 minutes (38.0, 60.5) in the first 10 cases to 34.5 minutes (31.5, 52.0) in the last 10 cases (*P* = .059).

**Conclusion:**

A standardized BiB-PFA workflow enables efficient, safe, and reproducible PVI with optional PWA and can be rapidly implemented during early operator experience.

**Key Findings**

▪ This study provides the first standardized, step-by-step workflow for pulmonary vein isolation (PVI) with optional posterior wall ablation (PWA) using a balloon-in-basket pulsed field ablation system.
▪ The integrated mapping-and-ablation design enables a catheter-only approach, supporting efficient and reproducible wide-antral isolation without the need for a separate mapping catheter.
▪ Procedures were characterized by short duration, low fluoroscopy time, successful acute PVI in all patients, and seamless integration of PWA without relevant prolongation.
▪ The workflow demonstrated a favorable acute safety profile and a rapid learning curve, with progressive reduction in procedure time during early operator experience.

## Introduction

Pulsed field ablation (PFA) is increasingly adopted as a catheter-based treatment modality for atrial fibrillation (AF), with a growing emphasis on single-shot technologies designed to improve procedural efficiency and reproducibility of pulmonary vein isolation (PVI).^1^ In this context, device architectures that integrate ablation and mapping into a single platform are of particular interest, given that they may reduce catheter exchanges, simplify workflows, and shorten procedural duration in routine clinical practice. The VOLT™ system (Abbott) is a next-generation PFA platform that integrates a balloon-in-basket (BiB) design with multipolar mapping capability, allowing circumferential lesion creation and real-time electrogram assessment within a single catheter.

The expandable BiB design promotes stable wall contact and homogeneous energy distribution, which may reduce shear stress, hemolysis, and inflammatory activation compared with alternative PFA designs or thermal energy sources.^2^, ^3^, ^4^, ^5^, ^6^ Early feasibility studies, including the multicenter VOLT-CE Mark study and the VOLT-AF investigational device exemption trial, demonstrated high acute PVI success rates and a favorable safety profile.^7^^,^^8^ However, standardized guidance for real-world implementation of the BiB-PFA workflow is lacking. Therefore, we present the worldwide first dedicated step-by-step procedural protocol for BiB-PFA, focusing on procedural standardization, safety, and workflow optimization in routine clinical practice.

## Methods

### Study population and trial design

This prospective, single-center, nonrandomized study enrolled consecutive patients with AF undergoing index PVI with or without additional posterior wall ablation (PWA) using the BiB-PFA system at the University Heart Center Lübeck, University Hospital Schleswig-Holstein. Procedures were performed as part of routine clinical care, and patients were prospectively enrolled in the institutional Lübeck Ablation Registry. Eligible patients were ≥18 years old with documented paroxysmal or persistent AF who provided a written informed consent. The exclusion criteria were active infection, a body mass index of ≥40 kg/m^2^, and stroke or myocardial infarction within the previous 3 months. The study was approved by the local ethics committee (WF-028/15) and conducted in accordance with the Declaration of Helsinki.^9^

### Preprocedural management

All patients underwent a standardized preprocedural evaluation according to institutional protocol. In individuals with elevated thromboembolic risk, transesophageal echocardiography was performed to exclude left atrial (LA) thrombus. Vitamin K antagonists were continued at a therapeutic international normalized ratio (2.0–3.0), whereas direct oral anticoagulants were withheld on the morning of the procedure.

### Sedation protocol

Sedation strategy—conscious or deep—was selected after structured counseling according to patient preference. Conscious sedation was not offered to patients with known anxiety or panic disorders. Deep sedation was performed with propofol, midazolam, and fentanyl. Conscious sedation omitted continuous propofol infusion to preserve partial responsiveness. In this setting, sedation and analgesia were achieved with metamizole, midazolam, fentanyl, and intravenous lidocaine, following our AWAKE protocol as described in detail in previously published work.^5^ All patients were continuously monitored using noninvasive blood pressure measurement, pulse oximetry, ECG, and capnography (capnography was used only in deep sedation). Sedation and analgesia were administered by specialized electrophysiology nursing staff under direct physician supervision.

### BiB preparation

**Figure 1:**
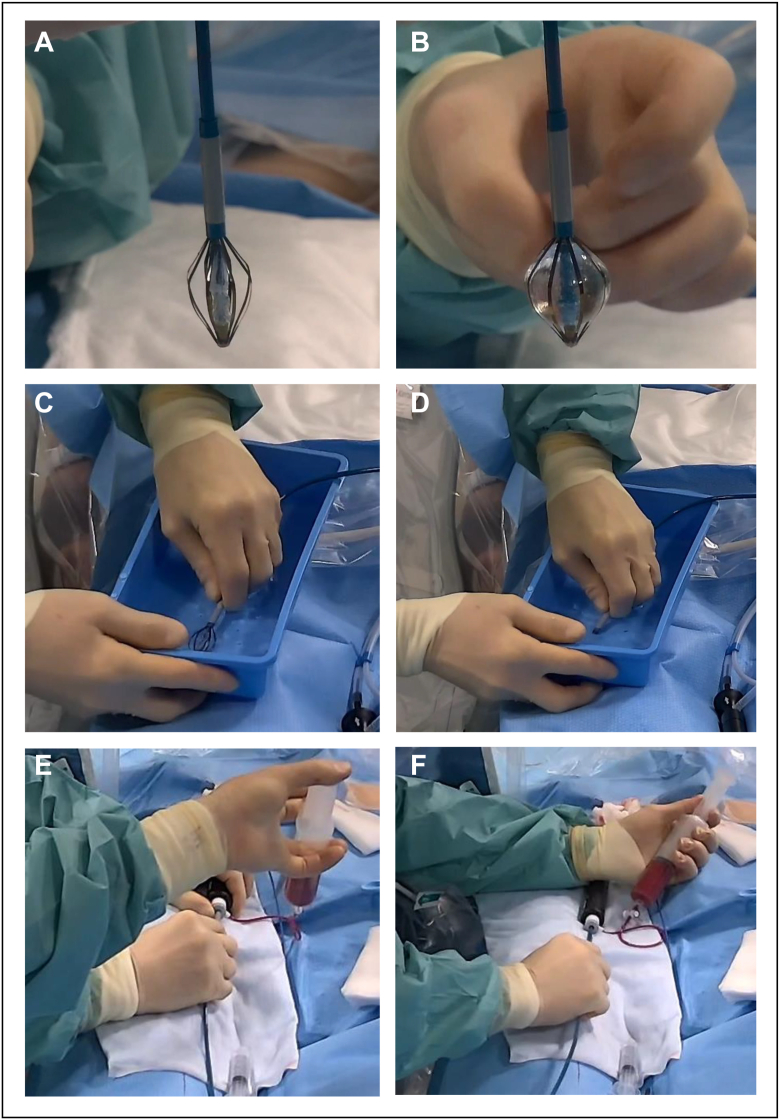
Balloon-in-basket (BiB) preparation and introduction protocol. **A** and **B:** Balloon preparation and preinflation to verify integrity and eliminate residual air. **C** and **D:** Advancement of the introducer aid over the balloon while submerged under water to ensure complete deairing and smooth assembly. **E** and **F:** Introduction of the BiB catheter through the steerable sheath under continuous saline infusion to maintain positive pressure and prevent air ingress.

The BiB-PFA system was prepared and preinflated before insertion to verify integrity, ensure proper function, and eliminate residual air. The introducer aid was retracted proximally, and the balloon was visually inspected for damage. For initial deairing, 1 mL of isotonic saline was drawn into a 10-mL syringe, connected to the balloon lumen, and negative pressure was applied for approximately 5 seconds; the 3-way stopcock was then closed. The syringe was subsequently disconnected, refilled with 10 mL of saline, and reattached to the balloon lumen. 10 mL of saline was injected and gently aspirated while the catheter was held in a downward orientation and lightly tapped to mobilize air (Figure 1A and 1B). Aspiration was repeated in the same position; during this step, approximately 7 mL entered the syringe while the remaining 3 mL stayed within the catheter owing to its internal volume. After closing the 3-way stopcock, the syringe was removed, refilled to 10 mL, and reconnected, and this aspiration–reinfusion cycle was repeated until no further air bubbles were visible. Once complete deairing was confirmed, the full 10 mL was aspirated, and the 3-way stopcock was closed. 1 mL of saline in the syringe was then replaced with 1 mL of contrast medium; the resulting 10-mL mixture was injected into the balloon 1 final time and aspirated again, and the 3-way stopcock was left open toward the syringe. The catheter was finally submerged in a sterile saline bath, and the introducer sheath was advanced over the balloon while submerged under water to eliminate any remaining air and facilitate smooth insertion (Figure 1C and 1D).

### Vascular access and transseptal puncture

Femoral venous access was obtained via 2 ultrasound-guided punctures using 8F sheaths. A diagnostic catheter was advanced into the coronary sinus under fluoroscopic guidance. Transseptal puncture was performed with an SL1 sheath (Abbott) and a BRK1 needle (Abbott) using the modified Brockenbrough technique. After successful LA access, intravenous unfractionated heparin was administered to maintain an activated clotting time of ≥300 seconds. Selective pulmonary vein (PV) angiography was performed using rotational angiography via the SL1 sheath (Abbott) to delineate PV anatomy and assess branching patterns. The transseptal sheath was subsequently exchanged over a guidewire for a steerable 13F delivery sheath (Agilis™ NxT, Abbott), which was advanced into the LA and initially positioned in the left superior PV (LSPV). The BiB-PFA catheter was introduced through the steerable sheath with continuous saline injection to maintain positive pressure and prevent air ingress (Figure 1C and 1D). The SL1 guidewire (Abbott) used during transseptal access was advanced through the lumen of the BiB catheter to facilitate stable engagement of the PVs. Balloon inflation was strictly performed outside the PV before gentle advancement toward the ostium.

### PVI workflow

PFA was delivered at a nominal voltage of 1800 V. For each PV, a structured 2-step lesion concept was applied:

1 2 ostial applications
2 1–2 antral applications

**Figure 2:**
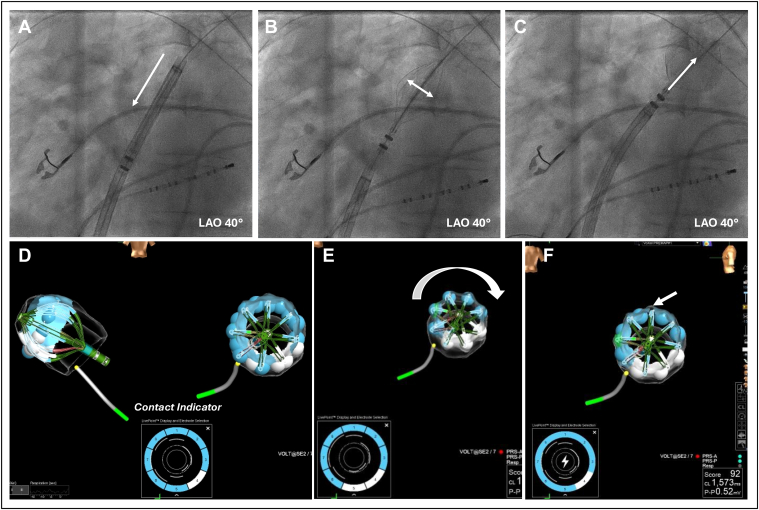
Balloon-in-basket (BiB) positioning and ablation of the LSPV. **A** and **B:** Advancement of the BiB catheter into the LSPV by withdrawal of the steerable sheath. **C:** Balloon inflation with 9 mL saline and 1 mL contrast. **D:** Energy delivery after confirmation of stable tissue contact (*blue indicator* = ≥2/3 electrode contact). **E** and **F:** Catheter rotation for the second ostial application to achieve circumferential coverage. LAO = left anterior oblique; LSPV = left superior pulmonary vein.

Between the 2 ostial applications, the BiB-PFA catheter was slightly rotated to improve interspline coverage and ensure circumferential lesion continuity (Figure 2E and 2F). The decision to perform 1 or 2 antral applications was guided by PV anatomy and electric field: in veins with larger ostia or when the antrum was not fully covered by the electric field with a single antral application, 2 antral applications were delivered to achieve wide-antral isolation.

Ablation was performed in a standardized sequence: LSPV, left inferior PV (LIPV), right superior PV (RSPV), and right inferior PV (RIPV). The BiB-PFA catheter was released at the antrum of the LSPV by withdrawal of the steerable sheath with the wire positioned in the LSPV (Figure 2A and 2B). The balloon was always inflated outside the vein and gently advanced to the ostium (Figure 2C). Adequate wall contact was verified using the tissue-contact indicator (Figure 2D). After the 2 initial ostial applications, 1 or 2 antral applications were performed. During antral positioning, the tissue-contact indicator was used to assess regional electrode–tissue interaction, and catheter orientation was adjusted as needed to ensure balanced circumferential coverage. Transition from the LSPV to the LIPV was achieved by retracting approximately 75% of the balloon into the steerable sheath, deflecting the sheath by ∼30 degrees, and performing a slight clockwise rotation. The guidewire was then advanced into the LIPV, followed by advancement and inflation of the BiB catheter.

In regions such as the carina, the tissue-contact indicator may not display a full blue signal despite adequate wall contact owing to anatomic constraints. The contact indicator is impedance based and reflects changes relative to baseline impedance, with an increase suggesting tissue contact. Given that the indicator requires ≥50% electrode–tissue contact to display a full signal, incomplete visualization does not necessarily indicate a lack of contact and should be interpreted in conjunction with impedance behavior and fluoroscopic catheter stability. Tissue contact was assessed qualitatively using the impedance-based tissue-contact indicator without applying a predefined quantitative cutoff (eg, no minimum number of electrodes required to display a full blue signal) before energy delivery.

Before ablation of the right-sided PVs, phrenic nerve capture was assessed by pacing from all splines at 20 mA/10 ms. If diaphragmatic capture occurred, ablation was performed using a reduced-energy setting (1400 V) with a minimum of 3 applications. Capture was assessed manually by direct palpation of the upper abdomen to promptly detect phrenic nerve capture.

**Figure 3:**
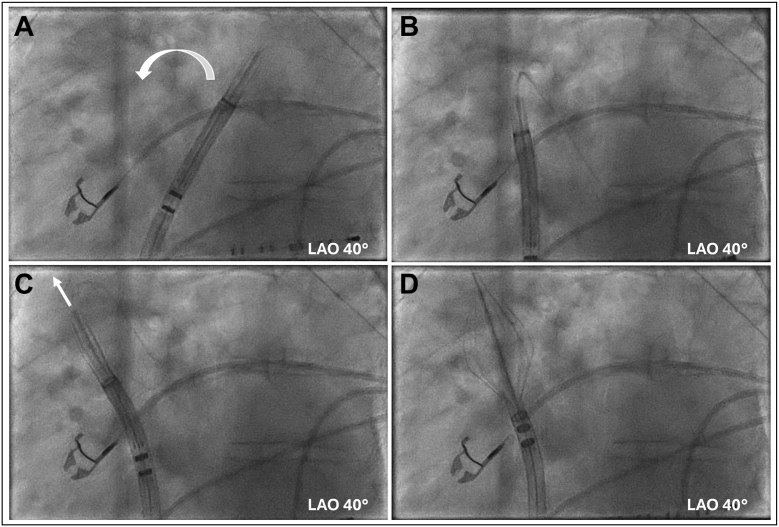
Access to the right superior pulmonary vein (RSPV). **A** and **B:** Sheath deflection (∼30°) and clockwise rotation under LAO fluoroscopy to align toward the RSPV. **C** and **D:** Wire advancement into the RSPV followed by balloon-in-basket catheter advancement and inflation. LAO = left anterior oblique.

**Figure 4:**
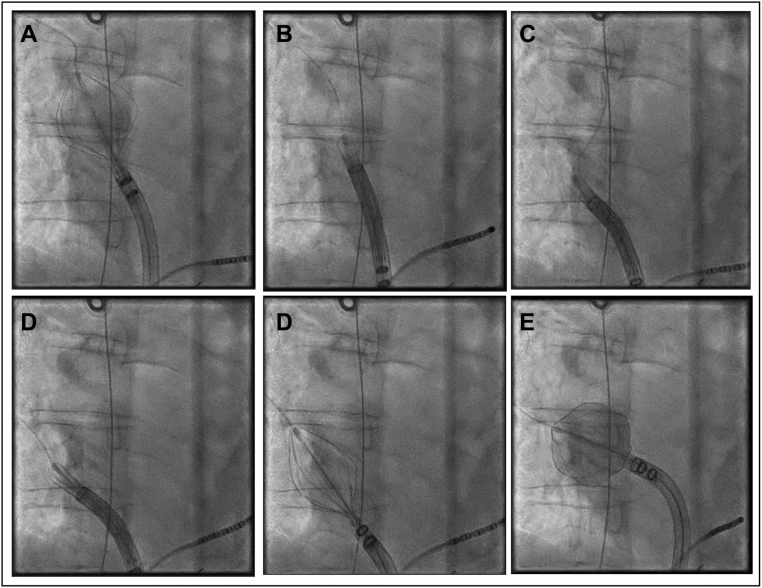
Access to the right inferior pulmonary vein (RIPV). **A** and **B:** Partial balloon retraction into the sheath. **C** and **D:** Increased sheath deflection (∼45°) with minimal counterclockwise rotation. **E** and **F:** Guidewire placement into the RIPV followed by balloon advancement, inflation, and positioning at the ostium.

Access to the RSPV was achieved by retracting the balloon approximately 75% into the sheath while maintaining the wire at least 30 mm beyond the sheath tip. The sheath was curved by ∼30 degrees and rotated clockwise under left anterior oblique (LAO) fluoroscopy until facing the 11-o’clock position in LAO 40° (Figure 3A and 3B). The guidewire was then advanced into the RSPV, followed by advancement and inflation of the BiB-PFA catheter (Figure 3C and 3D). Alternatively, the balloon was left inflated and fully outside the sheath, and the BiB catheter together with the sheath was gently rotated clockwise (Supplemental Figure 1). During this maneuver, the posterior wall was mapped in a fly-by fashion. Subsequently, without retracting the BiB catheter into the sheath, direct access to the RSPV was obtained under LAO 40° fluoroscopy by advancing the guidewire into the vein. Throughout this approach, the guidewire was continuously maintained at least 30 mm beyond the distal end of the BiB catheter to ensure atraumatic handling. For transition into the RIPV, the catheter was again fully deflated and retracted 75% into the sheath (Figure 4A and 4B). The sheath was deflected further to approximately 45 degrees with minimal counterclockwise rotation (Figure 4C and 4D). The guidewire was advanced into an upper branch of the RIPV, and the balloon was then advanced, inflated outside the vein, and guided toward the ostium (Figure 4E and 4F). Ablation was initiated at the ostium, followed by 2 antral applications using slight withdrawal and counterclockwise rotation to ensure complete PV coverage. Throughout all maneuvers, the guidewire was intentionally maintained slightly beyond the sheath tip to maintain an atraumatic leading edge during catheter manipulation.

### PWA

**Figure 5:**
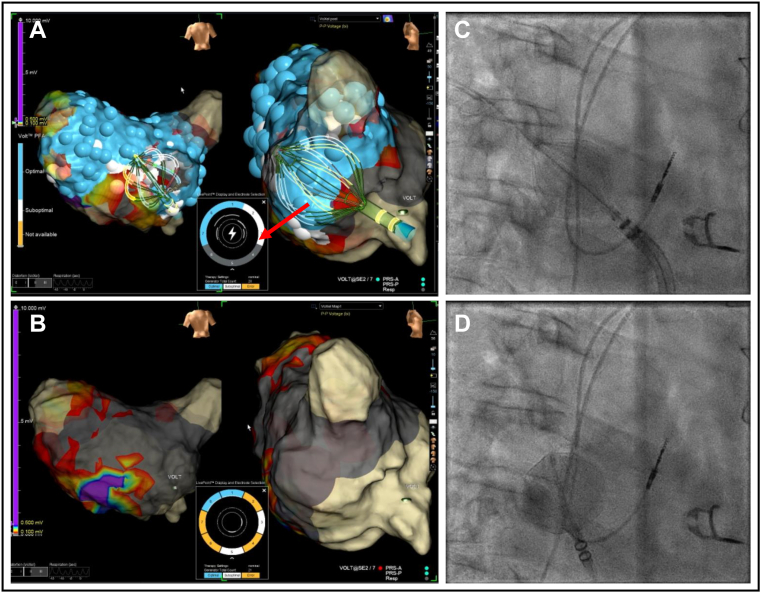
Posterior wall isolation using the balloon-in-basket catheter. The catheter is positioned along the left atrial posterior wall, and splines without adequate tissue contact are selectively deselected to optimize lesion delivery. **A:** Electroanatomic map during posterior wall ablation. **B:** Postablation electroanatomic map demonstrating posterior wall isolation. **C:** Corresponding fluoroscopic image in right anterior oblique 30° projection. **D:** Corresponding fluoroscopic image in left anterior oblique 40° projection.

PWA was performed in selected patients with posterior wall low voltage and/or persistent AF, with low voltage defined as either absent electrical activity or bipolar voltage amplitudes of ≤0.05 mV not distinguishable from noise or abnormal low-voltage areas with bipolar voltage amplitudes of ≤0.5 mV. The BiB-PFA catheter was positioned parallel to the posterior wall, facing the left PVs, and gently rotated clockwise to maximize posterior wall contact (Figure 5). To reduce catheter rigidity and improve conformity to atrial anatomy, the steerable sheath was intentionally kept slightly proximal to the balloon. Splines not contacting the posterior wall and oriented toward the roof or anterior wall were selectively deselected. Nominal energy (1800 V) was delivered using a minimum of 2 applications per region, with slight catheter rotation between applications to achieve contiguous lesion sets.

### Electroanatomic mapping

Electroanatomic mapping was performed exclusively with the BiB-PFA catheter, without the use of an additional dedicated mapping catheter given that mapping and lesion assessment were integrated into the ablation workflow. Lesion efficacy was assessed by reduction in signal amplitude and/or complete disappearance of local electrograms observed immediately during and directly after each energy application. After completion of PVI, a systematic remapping of all PVs was performed using the BiB-PFA catheter to confirm durable entrance block. Exit block testing by pacing was not routinely performed. No separate preprocedural electroanatomic reconstruction of the LA was performed before PVI. Instead, mapping was conducted dynamically during catheter manipulation. When transitioning from the left-sided to the right-sided PVs, the posterior wall was assessed using a “fly-by” mapping approach to identify residual electrical activity and evaluate lesion continuity. Systematic mapping of the anterior LA wall was not routinely performed.

### Postprocedural management

Hemostasis was achieved using vascular closure devices or figure-of-8 sutures with compression bandaging. The bandage was removed after 1–4 hours, and sutures were removed the next day. Transthoracic echocardiography was routinely performed immediately after the procedure, at 1 hour after procedure, and on postoperative day 1 to exclude pericardial effusion. Oral anticoagulation was resumed 6 hours after ablation and continued for at least 2 months, with long-term therapy guided by CHA_2_DS_2_-VA–based thromboembolic risk in accordance with current guidelines.^2^ A class IC or class III antiarrhythmic drug, as well as a proton pump inhibitor, was prescribed for 2 months.

### Statistical analysis

Continuous variables were tested for normality using the Shapiro–Wilk test and are presented as median [first quartile, third quartile]. Between-group comparisons were performed using independent-samples *t* tests or Mann–Whitney U tests, as appropriate. Categorical variables are reported as counts and percentages and compared using χ^2^ tests. Analyses were performed with IBM SPSS Statistics version 29.0.1.0 (IBM Corp, Armonk, NY), with 2-sided *P* < .05 considered statistically significant. A subgroup analysis compared procedural metrics between PVI alone and PVI plus PWA using the same statistical methods.

## Results

### Baseline characteristics

40 consecutive patients were enrolled. The study population had a median age of 64.5 years (56.5, 73.3); 41% were female, and 42.5% had paroxysmal AF. The median CHA_2_DS_2_-VA score was 2.0 (0.0, 3.0). Echocardiography showed a median LA volume index of 38.0 mL/m^2^ (25.8, 51.50) and a median left ventricular ejection fraction of 55.0% (55.0, 56.8). Additional baseline characteristics are presented in Table 1.

**Table 1:** Baseline characteristics of the study population

Variable	Value
Women	12 (30)
Age (y)	64.5 (56.5, 73.3)
BMI (kg/m^2)^	27.2 (24.8, 29.3)
Atrial fibrillation type	
Paroxysmal	17 (42.5)
Nonparoxysmal	23 (57.5)
CHA_2_DS_2_-VA	2.0 (0.0, 3.0)
Arterial hypertension	25 (62)
Diabetes	4 (10)
Coronary heart disease	7 (17.5)
Heart failure	10 (25)
Previous TIA/stroke	4 (10)
OSAS	2 (5)
Oral anticoagulation	34 (85)
Antiarrhythmic drug	5 (12.5)
LVEF (%)	55.0 (55.0, 56.8)
LAVI (mL/m^2^)	38.0 (25.8, 51.5)
NTproBNP (ng/L)	229.0 (92.0, 528.5)
Creatinine (μmol/L)	83.0 (72.6, 97.5)

Values are presented as median (first quartile, third quartile) or number (percentage), as appropriate.

AAD = antiarrhythmic drug; AF = atrial fibrillation; BMI = body mass index; LAVI = left atrial volume index; LVEF = left ventricular ejection fraction; NTproBNP = N-terminal pro-B-type natriuretic peptide; OAC = oral anticoagulation; OSAS = obstructive sleep apnea syndrome; TIA = transient ischemic attack.

### Procedural characteristics

AF was present at the procedure start in 37.5% of patients. Conscious sedation was used in 37.5% and deep sedation in 62.5%. The median procedural duration was 41.5 minutes (35.0, 59.0), with an LA dwell time of 27.5 minutes (25.0, 36.0) and a BiB-specific dwell time of 23.0 minutes (20.0, 25.8). The median fluoroscopy time was 6.8 minutes (5.2, 9.3). The complete Lübeck “How-To” workflow, including catheter preparation, transseptal access, structured lesion delivery, and PWA techniques, is presented in Supplemental Video 1. For PVI, a median of 4 applications was delivered across all veins, with reduced-energy delivery required in 30% of RIPV and 25% of RSPV. PWA was performed in 32.5% of patients, requiring a median of 5 applications (4, 50) and 3.0 deselected splines (3.0, 3.3), achieving acute posterior wall isolation in all patients. No major intraprocedural complications occurred. 1 femoral pseudoaneurysm required interventional treatment. Vascular closure devices were used in 32.5% of procedures. Procedural characteristics are presented in Table 2.

**Table 2:** Procedural characteristics of the study population

Variable	Value
AF at procedure	15 (37.5)
Sedation regimen	
Conscious	15 (37.5)
Deep	25 (62.5)
Procedural duration (min)	41.5 (35.0, 59.0)
LA dwell time (min)	27.5 (25.0, 36.0)
BiB LA dwell time (min)	23.0 (20.0, 25.8)
Fluoroscopy time (min)	6.8 (5.2, 9.3)
Contrast media (mL)	40 (40, 40)
LSPV	
Number of applications	4.0 (2.0, 4.0)
Reduced energy	0 (0)
LIPV	
Number of applications	4.0 (2.0, 4.0)
Reduced energy	0 (0)
RIPV	
Number of applications	4.0 (2.8, 4.0)
Reduced energy	12 (30)
RSPV	
Number of applications	4.0 (2.0, 4.0)
Reduced energy	10 (25)
Posterior wall (n = 13)	
Number of applications	5.0 (4.0, 5.0)
Reduced energy	0 (0)
PW acute block	13 (100)
Number of deselected splines	3.0 (3.0, 3.3)
Venous closure system	13 (32.5)
Major complications	
Pericardial tamponade	0 (0)
Phrenic nerve paralysis	0 (0)
TIA/stroke	0 (0)
Minor complications	
Groin-site bleeding	1 (2.5)
Transient ST-segment elevation	0 (0)

Values are presented as median (first quartile, third quartile) or number (percentage), as appropriate.

AF = atrial fibrillation; BiB = balloon-in-basket; LA = left atrium; LIPV = left inferior pulmonary vein; LSPV = left superior pulmonary vein; PW = posterior wall; RIPV = right inferior pulmonary vein; RSPV = right superior pulmonary vein; TIA = transient ischemic attack.

### Subgroup analysis: PVI alone vs PVI + PWA

Of the cohort, 27 patients (67.5%) underwent PVI alone and 13 (32.5%) underwent PVI + PWA. Persistent AF was present in 100% of patients undergoing PVI + PWA compared with 37.0% in the PVI-only group (*P* < .001). Procedural duration, LA dwell time, BiB dwell time, fluoroscopy time, and contrast volume did not differ significantly between groups (Table 3).

**Table 3:** Subgroup analysis comparing procedural metrics

Variable	PVI	PVI + PWA	*P* value
Number of patients	27 (67.5)	13 (32.5)	
Persistent AF	10 (37.0)	13 (100)	<.001
Procedural duration (min)	43.0 (34.5, 59.0)	40.0 (35.0, 43.0)	.874
LA dwell time (min)	29.5 (21.5, 36.8)	26.5 (26.0, 29.5)	.582
BiB LA dwell time (min)	22.0 (18.0, 27.5)	24.0 (22.5, 25.3)	.345
Fluoroscopy time (min)	7.2 (5.4, 9.4)	5.5 (5.2, 7.4)	.406
Contrast media (mL)	40.0 (40.0, 40.0)	40.0 (30.0, 40.0)	.203

Values are presented as median (first quartile, third quartile] or number (percentage), as appropriate.

AF = atrial fibrillation; BiB = balloon-in-basket; LA = left atrium; PVI = pulmonary vein isolation; PWA = posterior wall ablation.

### Subgroup analysis of sedation regimen

A subgroup analysis was performed to compare procedural characteristics between deep sedation (n = 25; 62.5%) and conscious sedation (n = 15; 37.5%). Trend to shorter procedural times was observed in the conscious sedation group. Procedural metrics did not differ significantly between both sedation strategies (Table 4).

**Table 4:** Subgroup analysis comparing sedation regimen

Variable	Deep sedation	Conscious sedation	*P* value
Number of patients	25 (62.5)	15 (37.5)	
PWA	8 (32.5)	5 (33.3)	.890
Procedural duration (min)	43.0 (35.0, 59.0)	39.5 (33.8, 55.5)	.467
LA dwell time (min)	29.5 (26.0, 36.5)	27.0 (22.0, 31.5)	.163
BiB LA dwell time (min)	24.5 (21.8, 27.0)	21.0 (17.5, 24.0)	.115

Values are presented as median (first quartile, third quartile) or number (percentage), as appropriate.

AF = atrial fibrillation; BiB = Balloon-in-basket; LA = left atrium; PWA = posterior wall ablation.

### Learning curve analysis

**Figure 6:**
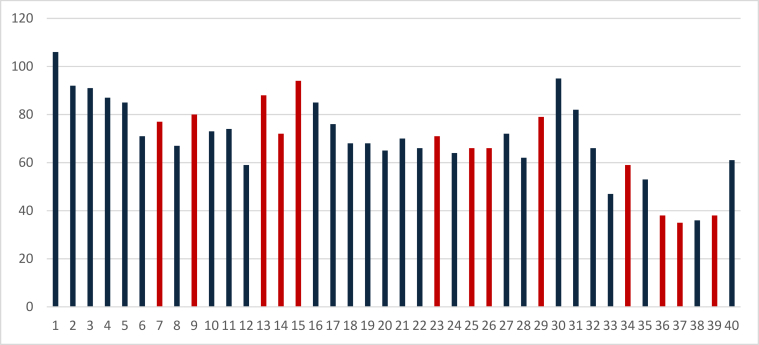
Learning curve displayed by procedural duration. Individual procedural durations for all 40 patients. Bars represent procedural time in minutes for each consecutive case. Patients who underwent PVI plus posterior wall ablation are shown in *red*, whereas patients who underwent PVI alone are shown in *blue*. PVI = pulmonary vein isolation.

Procedural duration decreased progressively across the first 40 consecutive cases, demonstrating a rapid learning curve for the BiB-PFA workflow (Figure 6). The mean procedural duration decreased from 50.0 minutes [38.0, 60.5] in the first 10 cases to 34.5 minutes (31.5, 52.0) in the last 10 cases (*P* = .059). All procedures were performed by a single experienced operator, minimizing interoperator variability and allowing focused assessment of workflow-related learning effects. Importantly, the observed reduction in procedural time occurred despite inclusion of PWA cases throughout the series (highlighted in *red*).

## Discussion

This prospective, single-center study provides the first systematic description of a standardized, step-by-step workflow for PVI with optional PWA using a BiB-PFA system in routine clinical practice. In a consecutive cohort, the protocol proved feasible, efficient, and acutely safe, with short procedures, consistent lesion delivery across all PVs, and successful PWA in selected patients. Importantly, these results were achieved without the need for an additional mapping catheter and with a rapid learning curve, underscoring the practicality of the proposed approach.

### Procedural performance and workflow

Overall procedural duration, LA dwell time, and fluoroscopy exposure were low despite the inclusion of patients with a substantial comorbidity burden and a predominance of nonparoxysmal AF. The structured 2-step lesion concept—comprising 2 ostial and 1–2 antral applications per vein—resulted in a uniform number of energy deliveries across all PVs and reproducible workflows independent of individual anatomy. The use of the BiB-PFA catheter as the sole mapping tool simplified catheter management and avoided catheter exchanges, which likely contributed to the observed efficiency. Although the integrated BiB-PFA catheter provides the key electrophysiological information required for procedural guidance—particularly regarding the presence or absence of low-voltage areas and fibrosis—it may be less sensitive for detecting very subtle conduction gaps or areas of slow conduction in complex atrial anatomies. Moreover, in contrast to cryoballoon systems, the higher stiffness of the BiB-PFA catheter allows controlled manipulation outside the sheath, facilitating task-specific maneuvers such as posterior wall fly-by mapping.

PWA could be seamlessly integrated into the same workflow. Despite being reserved for patients with persistent AF and/or posterior wall low voltage, PVI + PWA procedures showed comparable procedural and LA dwell times with PVI-only cases. This suggests that selective spline deactivation and targeted posterior wall positioning are technically straightforward once the basic PVI workflow is established and do not impose a major additional time burden.

### Safety considerations

No major intraprocedural complications occurred, including pericardial tamponade, stroke/transient ischemic attack, or phrenic nerve paralysis, and only 1 significant groin-site bleeding was observed. These findings align with previously reported safety data from the VOLT-CE Mark and VOLT-AF investigational device exemption trials and further findings from our group and support the favorable acute safety profile of the BiB-PFA system.^3^^,^^7^^,^^8^^,^^10^ The systematic use of phrenic nerve pacing with predefined energy reduction for right-sided veins represents an integral safety element of the workflow and may help mitigate the risk of phrenic injury observed with other PFA and thermal balloon technologies.^11^^,^^12^ Catheter exchanges through large-bore sheaths, particularly for separate mapping catheters, increase the risk of air embolism.^13^ The BiB-PFA system integrates mapping and ablation in a single catheter, thereby avoiding exchanges and potentially improving procedural safety.

### Learning curve and practical implementation

A key observation of this study is the rapid reduction in procedural duration across the first 40 cases, despite increasing case complexity through the incorporation of PWA. All procedures were performed by a single operator, making the observed learning curve directly attributable to progressive familiarity with sheath manipulation, balloon transitions between PVs, and posterior wall positioning. The early stabilization of procedural duration indicates that the standardized workflow can be internalized quickly, which is particularly relevant for centers transitioning from other PFA or thermal technologies.

These data support the notion that the BiB-PFA system is well suited for structured implementation programs and training curricula. The explicit description of catheter maneuvers—such as partial balloon withdrawal, sheath deflection angles, and rotation strategies—may further facilitate adoption by operators who are new to balloon-based PFA but experienced with cryoballoon techniques.

### Clinical implications

This standardized BiB-PFA workflow supports efficient real-world implementation of PVI with optional PWA. The integrated mapping-and-ablation design enables a catheter-only approach, reducing catheter exchanges and procedural complexity. A reproducible 2-step lesion concept facilitates consistent wide-antral PVI across varying anatomies, whereas PWA can be incorporated without a relevant increase in procedural duration. Compatibility with both deep and conscious, propofol-free sedation strategies further enhances flexibility of resource utilization.^5^

Safe application of the BiB-PFA workflow requires strict attention to several critical technical considerations:

1. Prevention of air embolism: continuous saline infusion during catheter introduction through the large-bore steerable sheath is essential to maintain positive pressure and prevent air ingress.
2. Avoidance of PV injury: balloon inflation must be performed strictly outside the PVs, followed by gentle advancement toward the ostium; intravein inflation may generate excessive pressure and increase the risk of venous injury or perforation.
3. Minimization of mechanical perforation risk: during all catheter manipulations, the guidewire should not be positioned only a few millimeters beyond the BiB tip, as this may create a rigid, penetrating leading edge. Instead, the guidewire should either be fully retracted within the BiB catheter or deliberately advanced at least 30 mm beyond the BiB tip to ensure a flexible, atraumatic configuration.

Awareness of these principles is particularly important during early operator experience and reinforces that procedural safety depends not only on device design but also on disciplined execution of standardized catheter handling techniques.

### Limitations

First, it reflects the experience of a single high-volume center and a single primary operator, which may limit generalizability to institutions with different workflows, operator experience, procedural volumes, or case mixes. Second, the sample size was modest, particularly for PWA, and the analysis was observational and nonrandomized; therefore, no causal inferences or comparisons with other ablation technologies can be made. Third, this study was designed to assess acute procedural performance and safety only. Lesion durability, long-term rhythm outcomes, and clinical follow-up were not systematically evaluated and were therefore beyond the scope of this methodological workflow report. Consequently, no conclusions can be drawn regarding long-term efficacy. Fourth, given the observational study design and the absence of a comparator group, no direct comparative conclusions regarding procedural performance or safety versus other catheter technologies or ablation modalities can be drawn. Finally, although a rapid learning curve was observed, prospective multicenter evaluations are required to confirm reproducibility across operators and clinical environments.

## Conclusion

This first dedicated workflow study of BiB-PFA demonstrates that a standardized, step-by-step protocol enables efficient, safe, and reproducible PVI with optional PWA in routine clinical practice. The combination of integrated mapping, a structured lesion strategy, and well-defined catheter techniques translated into short procedural times, a favorable safety profile, and a rapid learning curve. These findings support the adoption of the BiB-PFA workflow as a practical framework for contemporary AF ablation programs.

## Declaration of generative AI and AI-assisted technologies in the writing process

During the preparation of this work, authors used ChatGPT to improve language. After using this tool/service, the authors reviewed and edited the content as needed and take full responsibility for the content of the published article.

## Disclosures

R.R.T.: speaker (Pfizer, Abbott, Biosense Webster, Boston Scientific, Doctrina Med, cme4u, Medtronic, Radcliffe, and Wikonect), consulting/advisory (Boston Scientific, Biosense Webster, Capvision, Guidepoint, Haemonetics, Medtronic, Philips, and Abbott), institutional research (Biotronik, Abbott, Boston Scientific, Medtronic, LifeTech, and Johnson & Johnson), travel (Biosense Webster, Abbott, Boston Scientific, Medtronic, and Philips). S.H.: travel/educational grants (Abbott), educational grants (Johnson & Johnson). C.E.: research/travel grants and speaker (Abbott, Boston Scientific, LifeTech, Biosense Webster, CardioFocus, C.T.I. GmbH, and Doctrina Med). K.-H.K.: grants/personal fees (Abbott Vascular, Medtronic, and Biosense Webster). S.P.: travel/congress grants (LifeTech), educational/speaker grants (Abbott Medical), consultant (Active Health). J.-P.W.: funding (German Foundation of Heart Research F/29/19), speaker (Abbott and Doctrina Med), travel (Boston Scientific). The other authors have no conflicts of interest to disclose.

## Footnotes

Appendix Supplementary data associated with this article can be found in the online version at https://doi.org/10.1016/j.hroo.2026.02.022.

## References

[1] Reddy V.Y., Gerstenfeld E.P., Natale A. (2023). Pulsed field or conventional thermal ablation for paroxysmal atrial fibrillation. New Engl J Med.

[2] Wenzel J.P., Abdessadok R., Eitel C. (2025). Catheter design matters: hemolysis and renal function after pulsed field ablation with balloon-in-basket vs. Pentaspline systems. J Interv Card Electrophysiol.

[3] Wenzel J.P., Hatahet S., Abdessadok R. (2025). Differential inflammatory and myocardial biomarker response after pulsed field ablation for atrial fibrillation using balloon-in-basket versus Pentaspline catheter. Heart Rhythm.

[4] Wenzel J.P., Abdessadok R., Hatahet S. (2025). Balloon vs. balloon—comparison of hemolysis and renal markers after cryoballoon vs. ballon-in-basket pulsed field pulmonary vein isolation. Front Cardiovasc Med.

[5] Tilz R.R., Wenzel J.P., Eitel C. (2025). “Awake and ablated”: first experience with a balloon-in-basket PFA system without propofol. Heart Rhythm O2.

[6] Wenzel J.P., Hatahet S., Eitel C. (2026). Inflammatory and myocardial biomarker response following pulmonary vein isolation: cryoballoon versus balloon-in-basket pulsed field ablation. J Cardiovasc Electrophysiol.

[7] Lo M., Gambhir A., Sundaram S. (2025). Safety and effectiveness of a novel balloon-in-basket pulsed field ablation catheter for the treatment of paroxysmal and persistent AF: VOLT-AF IDE trial acute results. Heart Rhythm.

[8] Tilz R.R., Chierchia G.B., Gunawardene M. (2025). Safety and effectiveness of the first balloon-in-basket pulsed field ablation system for the treatment of atrial fibrillation: VOLT CE mark study 6-month results. Europace.

[9] World Medical Association (2013). World Medical Association Declaration of Helsinki: ethical principles for medical research involving human subjects. JAMA.

[10] Sanders P., Healy S., Emami M., Kotschet E., Miller A., Kalman J.M. (2024). Initial clinical experience with the balloon-in-basket pulsed field ablation system: acute results of the VOLT CE mark feasibility study. Europace.

[11] Heeger C.H., Sohns C., Pott A. (2022). Phrenic nerve injury during cryoballoon-based pulmonary vein isolation: results of the worldwide YETI registry. Circ Arrhythm Electrophysiol.

[12] Chéhirlian L., Koutbi L., Mancini J. (2025). High incidence of phrenic nerve injury in patients undergoing pulsed field ablation for atrial fibrillation. Heart Rhythm.

[13] Gier C., Simon E., Ahmed A. (2025). An ex vivo evaluation of air intrusion into pulsed field ablation sheaths during ablation and mapping catheter insertion. J Cardiovasc Electrophysiol.

