# Supplementary material for: Pulmonary vein isolation using a novel balloon-in-basket pulsed field ablation system: The Lübeck how-to protocol
Source: Heart Rhythm O2. 2026 Mar 6;7(6):1042–52. doi: 10.1016/j.hroo.2026.02.022 (PMC13307493; doi:10.1016/j.hroo.2026.02.022)
Supplement: Supplementary Material [file mmc1.docx]

**Supplements**

**

**Supplementary Figure 1. Alternative maneuver for transition from the left inferior pulmonary vein to the right superior pulmonary vein using the balloon-in-basket catheter.** With the balloon in-basket catheter left inflated and fully outside the sheath, the catheter–sheath assembly was gently rotated clockwise to perform posterior wall mapping in a fly-by manner. Direct access to the RSPV was then achieved under LAO 40° fluoroscopy without balloon retraction. The guidewire was maintained ≥30 mm beyond the distal end of the catheter to ensure atraumatic handling.

*Link for Video file.*

**Video 1.** Step-by-step pulmonary vein isolation and posterior wall ablation using the balloon-in-basket PFA system following the Lübeck protocol.
