# Supplementary figures and images for: Pulmonary vein isolation using a novel balloon-in-basket pulsed field ablation system: The Lübeck how-to protocol
Source: Heart Rhythm O2. 2026 Mar 6;7(6):1042–52. doi: 10.1016/j.hroo.2026.02.022 (PMC13307493; doi:10.1016/j.hroo.2026.02.022)

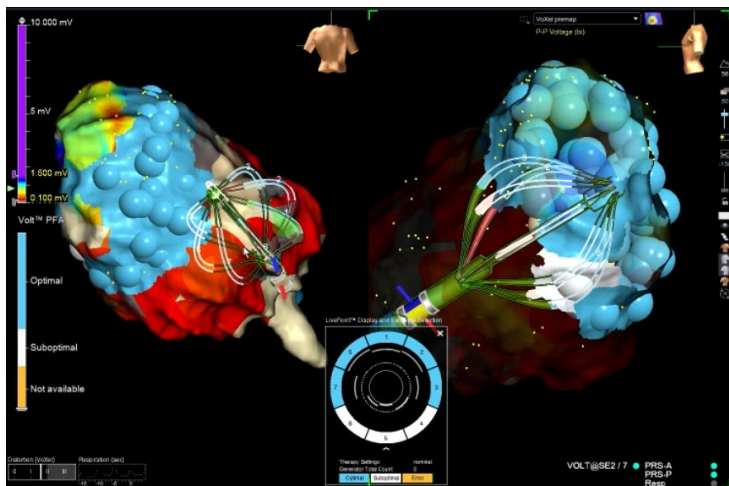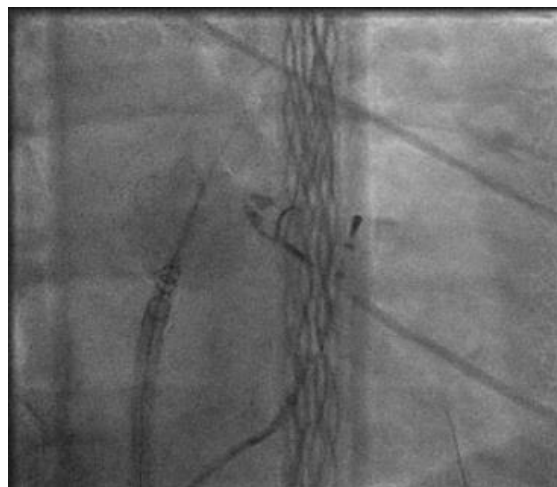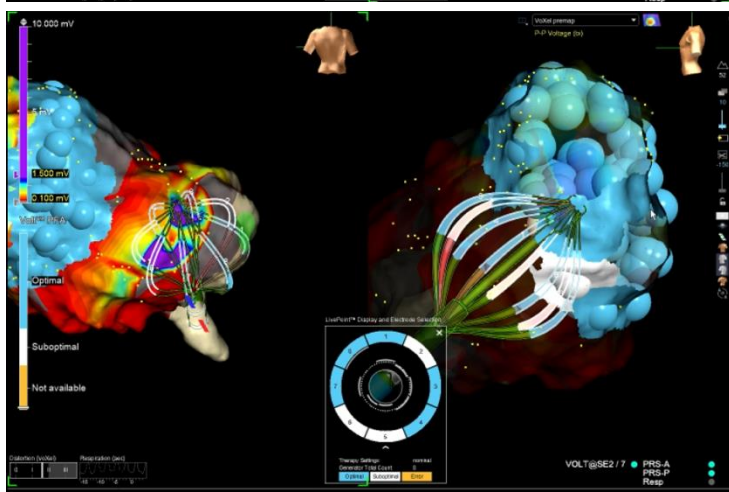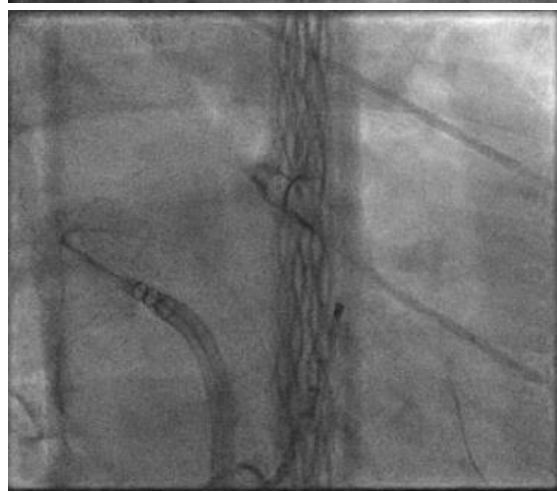

Supplement: Supplementary Figure [file mmc2.pdf]
